# The effect of intravenous iron supplementation compared to oral iron supplementation during pregnancy on neonatal outcomes—a systematic review of randomized controlled trials

**DOI:** 10.1007/s00431-025-06522-w

**Published:** 2025-10-13

**Authors:** Ilari Kuitunen, Kaisa Vepsäläinen, Laura Seppälä, Elli Toivonen, Atte Nikkilä

**Affiliations:** 1 Department of Pediatrics, Institute of Clinical Medicine, University of Eastern Finland, Kuopio, Finland; 2 Department of Pediatrics, Kuopio University Hospital, Puijonlaaksontie 2, Kuopio, 70210 Finland; 3 Department of Pediatrics, Tampere University Hospital, Wellbeing Services County of Pirkanmaa, Tampere, Finland; 4 Department of Gynecology and Obstetrics, Tampere University Hospital, Wellbeing Services County of Pirkanmaa, Tampere, Finland; 5 TamCAM - Tampere Center for Child, Adolescent and Maternal Health Research, Tampere University, Tampere, Finland

**Keywords:** Anemia, Iron deficiency, Oral iron supplementation

## Abstract

Anemia and iron deficiency during pregnancy have been associated with preterm birth and neonatal complications. However, the evidence on whether intravenous or oral supplementation should be used has been conflicting. The aim was to assess the effectiveness of intravenous iron compared to oral iron supplementation on objective neonatal outcomes. We conducted a systematic review and meta-analysis of randomized controlled trials. Two databases were searched in November 2024 and the search was updated in February 2025. The main outcomes were the rate of preterm birth, stillbirths, and neonatal mortality. Random-effect meta-analysis was used to calculate risk ratios (RR) with 95% confidence intervals (CI). Evidence certainty was assessed according to GRADE. A total of 375 studies were screened and finally 15 were included. Seven studies with 8431 pregnancies analyzed the risk of preterm birth, and the risk appeared to be similar in both groups (RR 0.96, CI 0.86 to 1.07; moderate certainty evidence). Five studies with 8639 pregnancies analyzed the risk of stillbirth and found no difference (RR 0.85, CI 0.64 to 1.13; low certainty evidence). The neonatal mortality rate was 2.0% in the intravenous iron group, and 2.3% in the oral iron group (RR 0.90, CI 0.66 to 1.22; low certainty evidence). Cord hemoglobin levels were comparable between the study groups (mean difference 0.05 g/l, CI −0.33 to 0.24; low certainty evidence), and ferritin levels were slightly higher in the intravenous group (mean difference 19 µg/l, CI 0.5 to 38; low certainty evidence). *Conclusions*: Neonatal clinical outcomes did not differ between intravenous and oral iron supplementation treatment in pregnancy. A higher ferritin level in umbilical cord blood was found in the intravenous iron supplementation group, but the clinical relevance of this difference is unknown. Based on the results of this study, oral iron supplementation is a sufficient way to treat maternal iron deficiency, when focusing on objective neonatal outcomes.

*Trial registration*: PROSPERO 2024 CRD42024615533 Available from: https://www.crd.york.ac.uk/prospero/display_record.php?ID=CRD42024615533.

**Table:** 

**What is Known:** • *Iron-deficiency anemia during pregnancy is associated with preterm birth and neonatal complications.* • *Intravenous iron increases maternal hemoglobin faster than oral iron, but benefits for neonatal outcomes have been unclear.*
**What is New:** • *This systematic review and meta-analysis shows that neonatal outcomes—including preterm birth, stillbirth, and mortality—do not differ between intravenous and oral iron supplementation.* • *Intravenous iron yields higher cord ferritin, but its clinical relevance remains uncertain.*

**Supplementary Information:**

The online version contains supplementary material available at 10.1007/s00431-025-06522-w.

## Introduction

Iron-deficiency anemia (IDA) during pregnancy is a common and global issue. The prevalence of iron-deficiency anemia has varied between 25 and 40% [1], whereas the prevalence of iron deficiency without anemia (ID) has been estimated to be 40–50% during the first trimester [2]. Maternal anemia has consistently been associated with obstetric and neonatal complications, such as preterm birth, low-birth weight, and intrauterine growth retardation [3–5]. Daily oral iron administration during pregnancy has been shown to reduce maternal anemia, but the impact on neonatal outcomes has been very low of certainty [6]. Follow-up studies have associated maternal anemia to offspring psychologic development and psychiatric wellbeing [7, 8].

The significance of iron deficiency without anemia (ID) is less clear. Iron demand increases during pregnancy due to fetal and placental growth and increased erythrocyte production. The interaction between the placenta and fetus in maternal IDA/ID is not well understood. On one hand, it seems that the placenta prioritizes fetal iron over maternal iron, and the correlation between maternal iron status and offspring iron status exists when maternal ID/IDA is severe [9]. On the other hand, it seems that maternal conditions causing iron deficiency, such as diabetes, can cause iron deficiency in the offspring [10]. Excessive iron intake in pregnancy seems also problematic, increasing the risk for preterm birth and stillbirth [11].

Several guidelines recommend screening for anemia or even universal iron supplementation in pregnancy [12–14], although the benefits of neither iron supplementation nor iron-deficiency screening are well established [15]. Iron supplementation is most often administered orally in both prophylaxis and treatment of IDA, as oral preparations are widely available, easy to self-administer, and affordable. In the treatment of IDA, intravenous preparations increase maternal hemoglobin more rapidly and to higher levels than oral supplementation, but the reduction of anemia-related complications is uncertain [16]. Previous studies have reported that neonatal outcomes of mean birthweight and mean hemoglobin levels have been similar between oral and intravenous iron supplementation strategies, but an overall review on clinical outcomes has been lacking [17, 18].

The aim of our study was to systematically compare the effect of intravenous iron supplementation to oral iron supplementation during pregnancy on neonatal outcomes.

## Methods

### Search process and data extraction

We searched PubMed and Scopus databases for this systematic review and meta-analysis. The search was conducted originally on November 15, 2024, and updated on February 10, 2025. The complete search strategy is provided in the supplementary materials. The search results were uploaded to Covidence software (Veritas Healthcare Inc, Melbourne, Australia), where automation tools removed duplicates. Then we used the automated Cochrane’s randomized trial filter to exclude clear non-randomized studies before screening. The automated tool has been proven to have high specificity [19]. Finally, two authors performed independently abstract screening, and full-text screening in the Covidence software. Conflicting classifications were solved by a mutual decision. Data extraction was performed by one author to a pre-designed Excel spreadsheet. Another author validated and checked the data for errors. The following information was extracted from each included study: bibliometric information, study setting, country, intervention description, comparator description, outcome definitions, trial characteristics, blinding, funding, conflicts of interest.

### Inclusion and exclusion criteria

We included parallel-grouped individually randomized controlled trials. The intervention was required to be an administration of intravenous iron supplementation during pregnancy and the comparator was required to be per orally taken iron supplementation. Thus, studies which compared two different intravenous iron supplementations were not included. The included studies needed to report at least one neonatal outcome, and studies which reported only maternal outcomes were not included. Non-randomized studies, reviews, and conference abstracts were excluded. Non-English written studies were included if found and translated by automatic translation tools.

### Main outcomes

We focused on three main outcomes. The first was the proportion of preterm births, and preterm birth was classified as a birth occurring before the gestational week 37 + 0. The second outcome was the perinatal mortality rate. Perinatal mortality includes stillbirths and neonatal deaths up to 28 days of age. The third main outcome was the need for neonatal intensive care unit treatment during the initial hospital stay.

Our secondary outcomes were Apgar-scores at 1 min and 5 min, neonatal hemoglobin levels and ferritin levels measured either from cord blood samples or during the neonatal period (first 28 days of life), birth weight, and neonatal diagnoses.

### Evidence appraisal

Risk of bias for the included studies was assessed with the Cochrane’s risk of bias 2.0 tool, and it was assessed separately for subjective and objective outcomes [20]. Two authors performed the assessments independently and conflicting cases were solved by reaching a mutual consensus. Evidence certainty for each outcome was assessed according to the Grading of Recommendations Assessment, Development and Evaluation (GRADE) framework [21]. In the GRADE assessment, we considered a relative difference of more than 10% as clinically relevant when considering the imprecision to the estimates. This approach was selected by discussing the minimally contextualized approach to the main outcomes [22]. We did not use this definition to pregnancy duration, where we considered an absolute difference of 1 week as a meaningful difference.

### Statistical analysis

Analyses were performed in the RevMan web interface, and we followed the guidance of the Cochrane Handbook in the analysis method selection [23]. We used random-effects DerSimonian and Laird inverse variance method to calculate pooled risk ratios (RR) with 95% confidence intervals (CI) for categorized outcomes. Absolute treatment effects per 100 or 1000 patients were calculated with CI for all main outcomes. For continuous outcomes, we transformed the measures to the same scale (hemoglobin g/dl, and ferritin µg/l) and used inverse variance random-effect meta-analysis to calculate mean differences (MD) with CI.

Subgroup analyses were performed based on the geographic locations and anemia status, as these cause heterogeneity due to genetic and dietary variations. Furthermore, we performed a sensitivity analysis where studies with only low risk of bias were included. Publication bias was analyzed by visual examination of funnel plots, when at least five studies analyzed the same outcome [24]. Statistical heterogeneity was assessed by examining the *I*^2^ statistic, and when statistical heterogeneity was high (> 40%), it influenced the evidence certainty assessment. However, the decision to use random-effect models in meta-analysis was made by a prior assumption of clinical heterogeneity in the patient populations.

We have reported our study according to the Preferred Reporting Items in Systematic Reviews and Meta-Analyses (PRISMA) guidelines and have provided the checklist in the Supplementary materials [25].

## Results

### Search results and study characteristics

A total of 996 reports were uploaded to Covidence, where duplicates (*n* = 337) and non-randomized studies (*n* = 284) were excluded automatically. After screening 375 studies, 34 studies were further assessed. Finally, 15 studies were included [26–40] (Figure S1). All studies were non-blinded randomized trials and conducted between 2002 and 2024 (Table S1). Of the studies, five were from high-, six from middle-, and three from low-income countries (Table S1). Fourteen of the 15 studies included anemic patients, of which nine focused on iron-deficiency anemia. The number of included participants varied from 47 to 4699 pregnant women (Table S2). Mean ages and parity were comparable between the intravenous and oral iron groups in all studies. Mean hemoglobin and ferritin levels had clear variation between the studies, but in-study comparisons showed that the intravenous and oral iron groups were comparable (Table S2). Funding information was mostly reported and two of the included trials were sponsored by a pharmaceutical company. Conflicts of interest were properly reported in 11 of the 15 studies (Table S2).

### Risk of bias

The risk of bias for objective outcomes was overall low in seven studies, had some concerns in three studies, and was high in five studies. Most of the issues rose from improper randomization and from the lack of pre-registration or statistical analysis plan (Figure S2). For subjective outcomes, the overall risk of bias was low in seven studies, had some concerns in two studies, and was high in six studies. Most of the downgrades came from the measurement of the outcome as the studies were not blinded and if the outcome assessment was described insufficiently (Figure S3).

### Gestation length and preterm birth

**Fig. 1:**
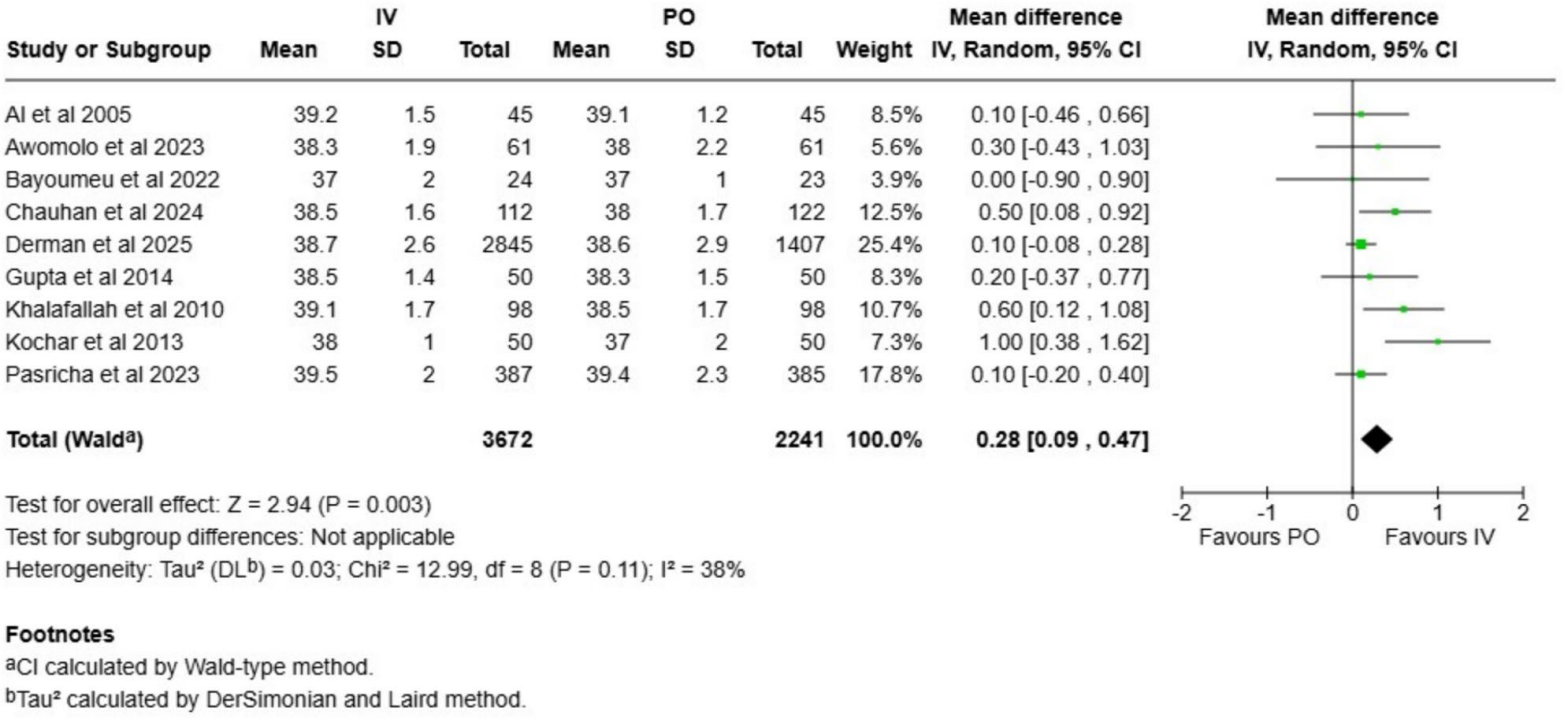
Forest plot of the comparison of mean gestational weeks at the time of the delivery between intravenous and oral iron supplementation group

A total of nine studies with 5913 neonates reported the mean gestation length and the mean difference was 0.3 weeks longer (CI 0.1–0.5) in the intravenous iron group (Fig. 1). We rated the evidence certainty as moderate (Table 1). When only low risk of bias studies were included, the estimate was similar 0.35 weeks (CI 0.02–0.69; Figure S4). In the stratified analysis, the effect was similar both in LMIC and high-income countries (Figure S5). We did not detect signs of publication bias (Figure S6).

**Table 1:** Summary of findings and evidence certainties assessed according to GRADE framework

Outcome	*N* of studies	*N* of participants	Absolute risk		Relative effect (CI)	Absolute effect (CI)	GRADE
			**Intravenous**	**Oral iron**			
Gestation length	9	5913	Not applicable	Not applicable	MD 0.3 weeks (0.1 to 0.5)	Not applicable	Moderate^a^
Rate of preterm births	7	8431	13 per 100	14 per 100	RR 0.96 (0.86 to 1.07)	1 less per 100 (2 less to 1 more)	Moderate^a^
Stillbirth rate	5	8639	20 per 1000	24 per 1000	RR 0.85 (0.64 to 1.13)	4 less per 1000 (9 less to 3 more)	Low^b^
Neonatal mortality rate	5	8312	20 per 1000	23 per 1000	RR 0.90 (0.66 to 1.22)	3 less per 1000 (8 less to 5 more)	Low^b^
Birthweight	13	6887	Not applicable	Not applicable	MD 15 g (−7 to 37)	Not applicable	Moderate^a^
Cord hemoglobin	8	4527	Not applicable	Not applicable	MD −0.05 g/l (−0.33 to 0.24)	Not applicable	Low^c^
Cord ferritin	8	4699	Not applicable	Not applicable	MD 19 µg/l (0.5 to 38)	Not applicable	Low^c^

*MD* mean difference, *RR* risk ratio, *CI* confidence interval

^**a**^Downgraded once due to risk of bias

^b^Downgraded once due to risk of bias and once due to imprecision

^c^Downgraded once due to risk of bias and once due to inconsistency

**Fig. 2:**
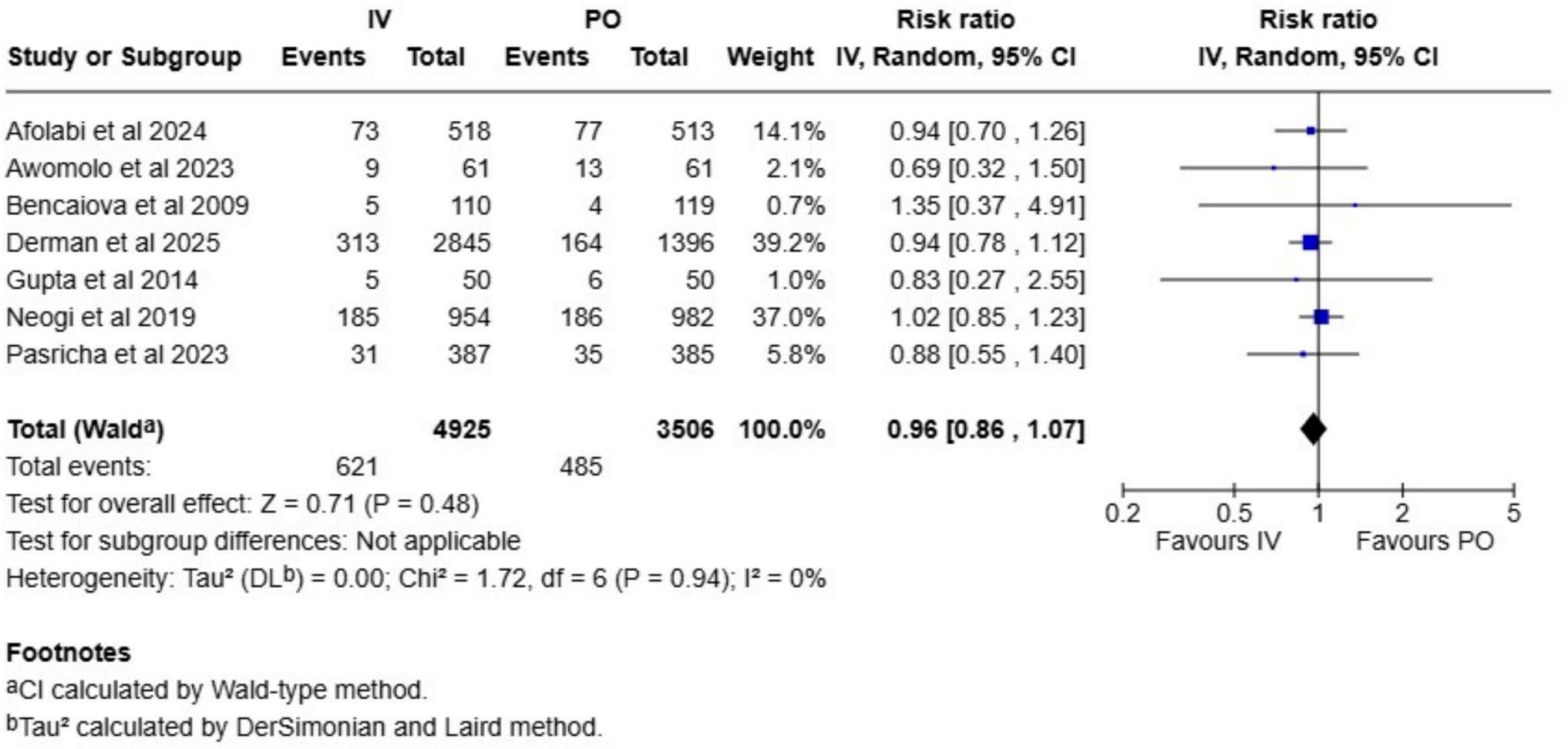
Rate of preterm birth compared between intravenous and oral iron supplementation group

Preterm birth rate was analyzed in seven studies with 8431 deliveries, and the rate was 13% in the intravenous iron group and 15% in the oral iron group, RR 0.96 (CI 0.86–1.07; Fig. 2). We ranked the evidence certainty as moderate. (Table 1) In the sensitivity analysis with only low risk studies included, the effect estimates remained unchanged (RR 0.97, CI 0.86–1.08; Figure S7). The stratified analysis did not show a difference between LMIC and high-income countries (Figure S8). We did not detect evidence of publication bias in the funnel plot (Figure S9).

### Stillbirths and neonatal mortality

**Fig. 3:**
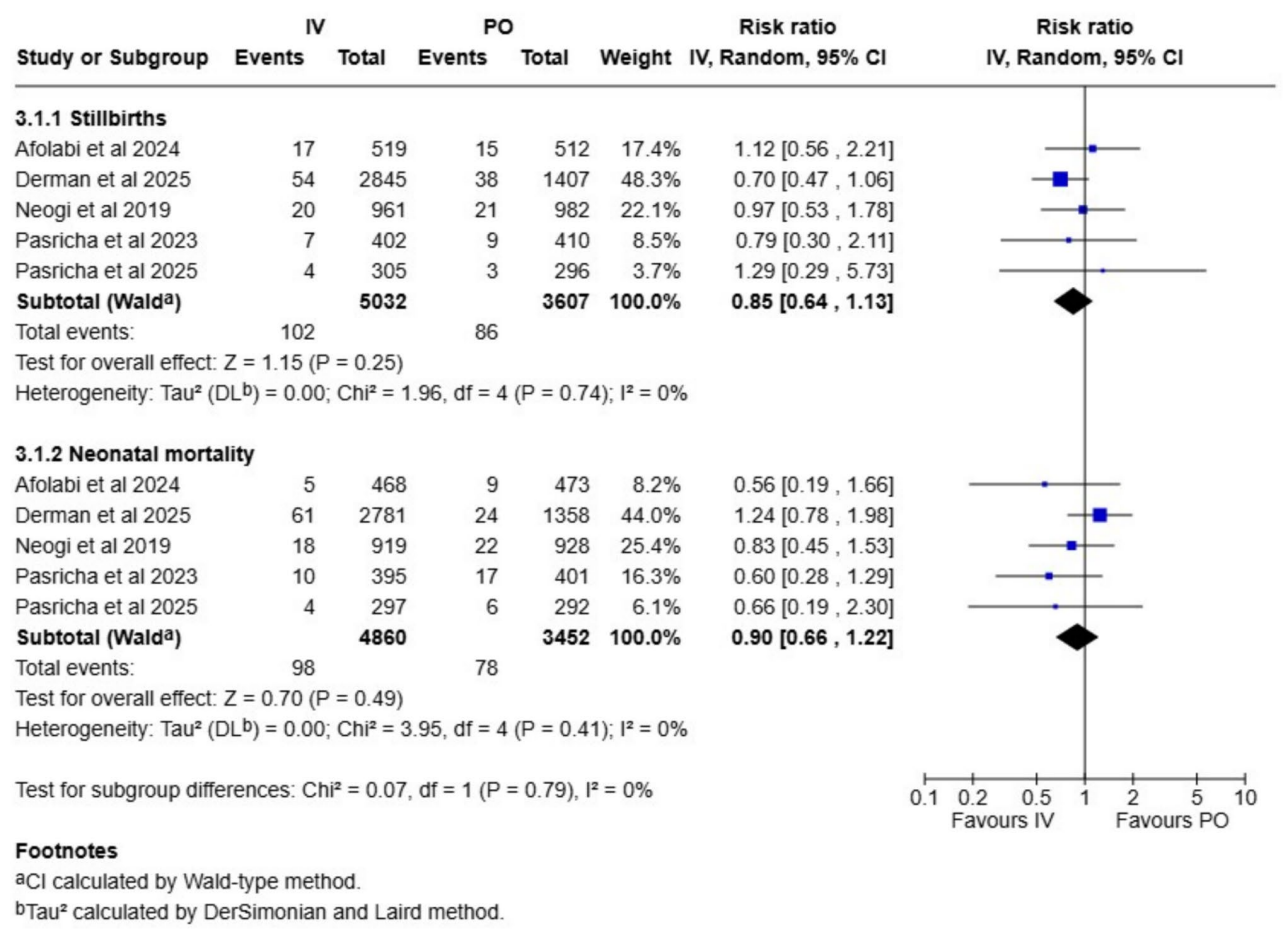
Stillbirth rate and neonatal mortality rate compared between intravenous and oral iron supplementation group

Five studies with deliveries conducted in LMIC countries analyzed both stillbirths and neonatal mortality rate. The stillbirth rate was 2.0% in the intravenous iron group and 2.4% in the oral iron group (RR 0.85, CI 0.64–1.13; Fig. 3). We ranked the evidence certainty as low (Table 1). The neonatal mortality rate was 2.0% in the intravenous iron group and 2.3% in the oral iron group (RR 0.90, CI 0.66–1.22; Fig. 3). We ranked the evidence certainty as low. Due to the low number of included studies publication bias was not assessed.

### Secondary outcomes

**Fig. 4:**
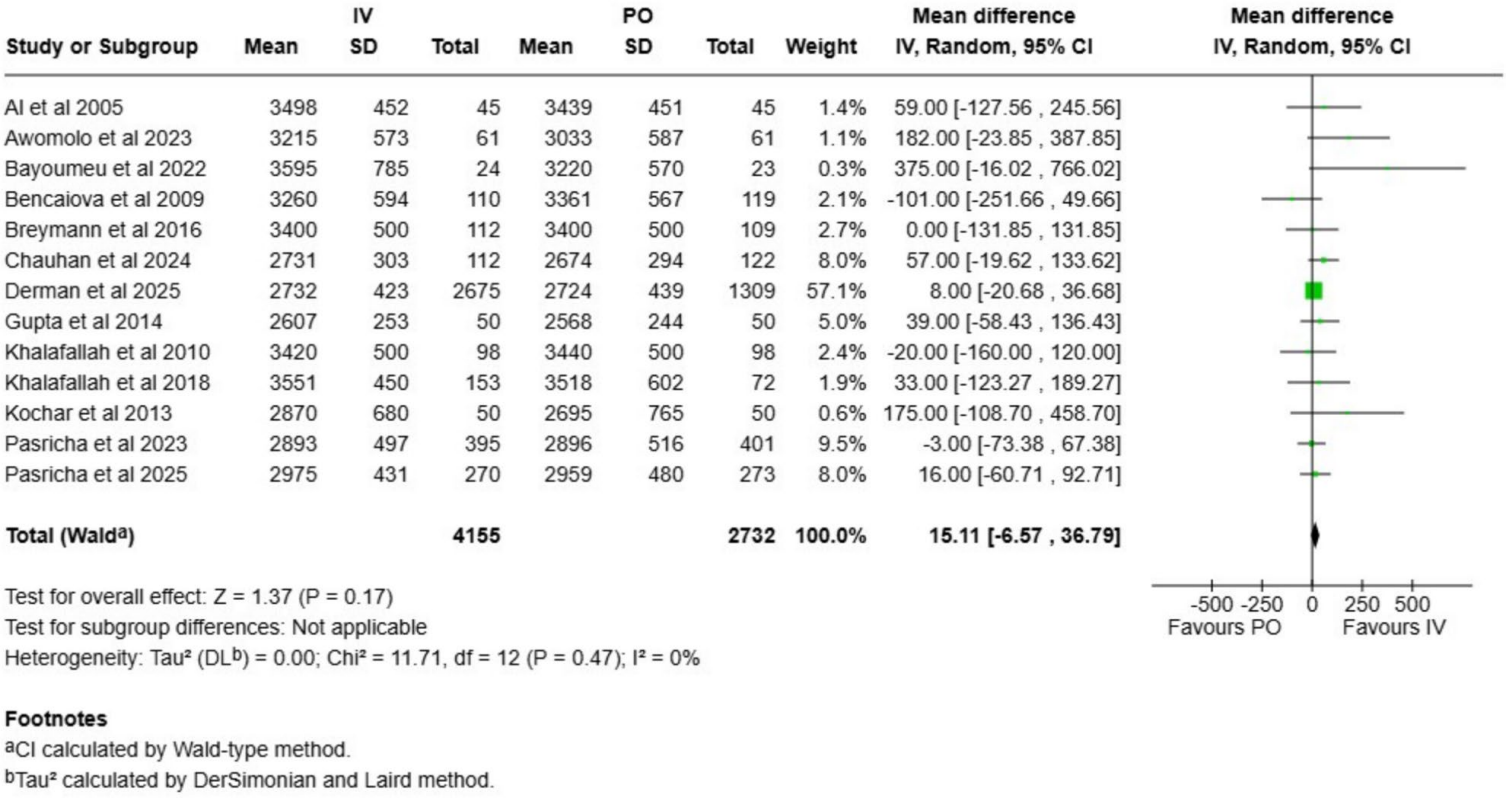
Birth weight compared between intravenous and oral iron group

Birthweight was assessed in 13 trials with 6887 neonates. The mean difference was 15 g (CI −7 to 37 g; Fig. 4). We ranked the evidence certainty as moderate (Table 1). In the sensitivity analysis with only low risk of bias studies included, the difference remained minimal (7 g, CI −17 to 32 g; Figure S10). In the stratified analysis, there was no evidence of a difference either in LMIC or high-income countries (Figure S11). No signs of publication bias were detected (Figure S12).

**Fig. 5:**
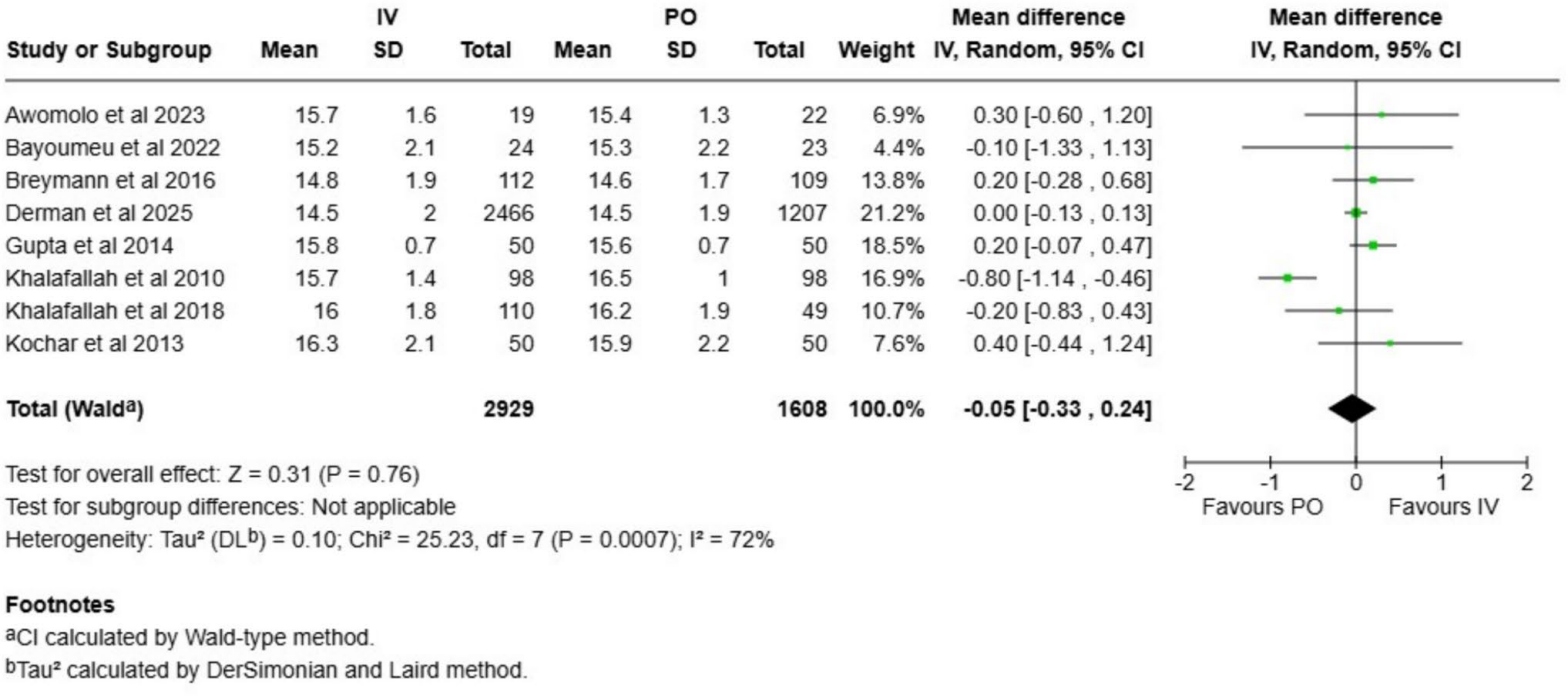
Cord blood hemoglobin measurements compared between intravenous and oral iron supplementation group

**Fig. 6:**
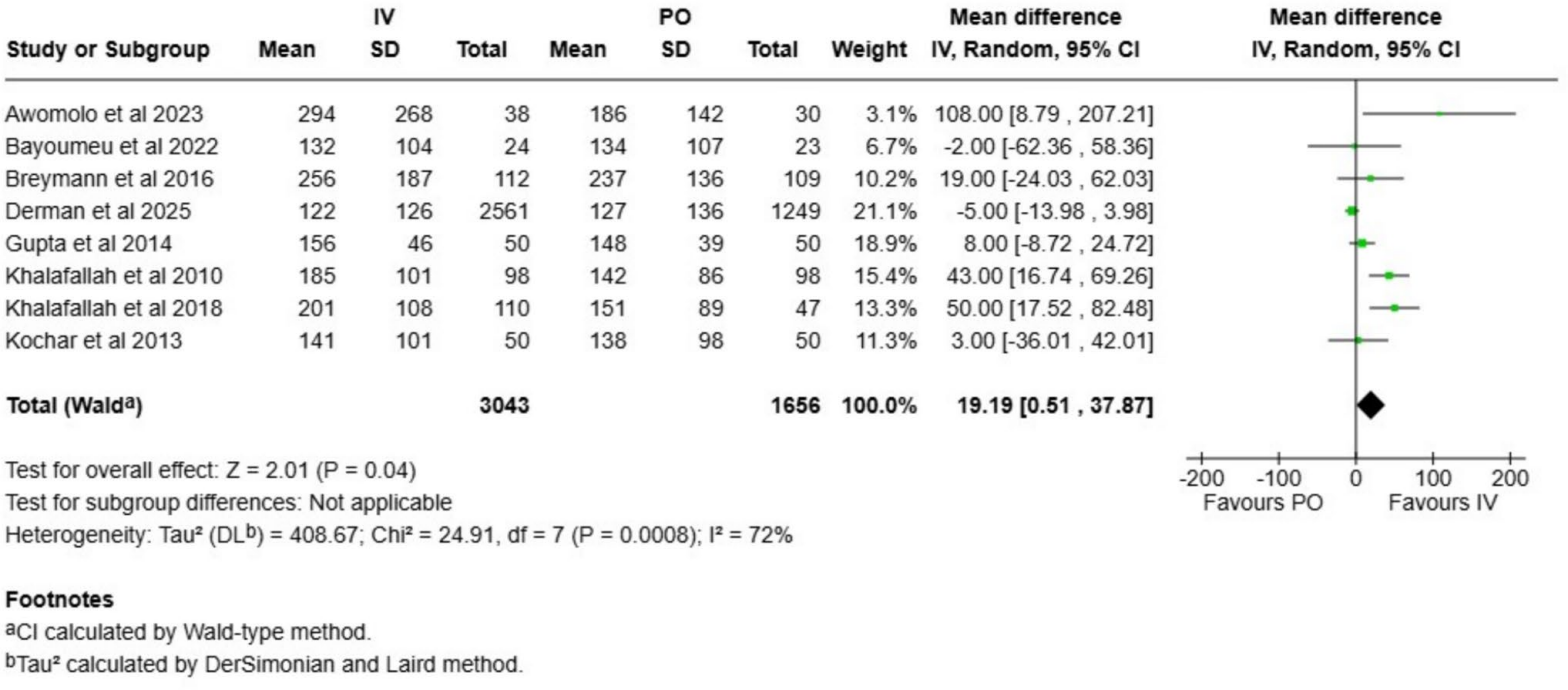
Cord blood ferritin measurements compared between intravenous and oral iron supplementation group

Cord hemoglobin and ferritin levels were analyzed in eight studies. The mean difference in hemoglobin level was −0.05 g/dl (CI −0.33 to 0.24 g/l; Fig. 5), and the mean difference in the ferritin level was 19 µg/l (CI 0.5 to 38; Fig. 6). Evidence certainty was ranked as low for both these outcomes (Table 1). In the sensitivity analysis with only low risk of bias studies included, the effect estimates changed for hemoglobin (MD −0.33, CI −0.91–0.25; Figure S13), and for ferritin (MD 27, CI −13–68; Figure S14). Both outcomes did not show signs of publication bias (Figures S15–16).

## Discussion

### Main findings

Overall, we found low to moderate certainty evidence that intravenous iron supplementation did not provide benefits over oral iron supplementation for clearly objective neonatal outcomes of preterm births, stillbirths, or neonatal mortality. According to low certainty evidence cord hemoglobin levels were comparable between the treatment strategies, but cord ferritin levels were higher in intravenous treatment groups.

### Comparison to previous literature

Our findings are in line with some of the previous meta-analyses: intravenous and oral iron therapy do not differ considering neonatal clinical outcomes [16–18]. The rates of preterm birth or low-birth weight neonates have been comparable in all previous meta-analyses, which have included up-to eight studies. These three prior meta-analyses have consistently shown that birthweight is approximately 50–70 g higher in the intravenous iron group, which aligns exactly to the estimate of this review [16–18]. All three of these prior meta-analyses reported that cord hemoglobin levels were comparable. Only one prior meta-analysis from 2019 had analyzed the cord ferritin level, and found no difference [17], whereas our current review found that the ferritin levels were higher in the intravenous treatment group.

Intravenous treatment seemed to in higher cord ferritin levels. Prior literature on the association of cord ferritin levels and infant outcomes is vague. A recent Canadian study showed that the mean ferritin levels increase by 6 µg/l per week until term weeks and then start to decrease by 2–3 µg/l per week [41]. The median cord ferritin was 100 in their sample. The mean ferritin levels in all of the included studies in this review were above this, indicating that the iron supplementation has been effective. A follow-up study of one of the included studies in this review was published recently [29], where they expanded the hematological parameter follow-up until 6 months of age [42]. They did not find any differences in the infant hemoglobin or ferritin levels at any time point between the orally and intravenously treated.

Most of the studies estimating the long-term health outcomes in IDA or ID have used the maternal ferritin levels as exposure. However, studies have not demonstrated a clear association between maternal ferritin levels to infant ferritin levels [43]. There are only a few studies which have used the cord ferritin levels as the exposure in the analyses. One of the studies found an association between low cord ferritin levels and worse neurodevelopmental outcomes [7]. The cutoff in their analyses was 76 µg/l for the cord ferritin. Again, in this current review, all of the included studies had a mean ferritin level of 134 µg/l or more. However, it could be speculated, whether the cord ferritin level could be caused by placental insufficiency, which then could lead to deficiencies in other important nutrients [44]. The association between iron status and worse outcomes in children have come from studies where the IDA has not been treated well. Currently, it seems that both oral and intravenous treatment treat the IDA well, and the cord ferritin levels in this review were notably higher in both groups than in the risk studies. Thus, the found difference of 19 µg/l in cord ferritin could be interpreted as non-clinically significant, as both strategies have increased the ferritin levels from the iron-deficiency levels at the start of the interventions.

### Strengths and limitations

A limitation of our review is that the studies did not report all the outcomes that we had planned to analyze in our protocol. The need for neonatal intensive care unit admissions was not reported, and similarly, there were no follow-up hemoglobin or ferritin measurements of the neonates after the discharge from birth hospital. Furthermore, the included studies had generally some concerns or a high risk of bias which lead to downgrading of evidence certainties. Furthermore, generally and globally the most important outcomes of stillbirths and neonatal mortality were analyzed only in three studies. We had no major protocol violations, which can be seen as a strength of this review.

### Implications for clinical practice and future studies

Intravenous iron supplementation during pregnancy did result in similar neonatal outcomes as oral iron supplementation. Intravenous iron therapy demands more resources, and our results provide important information for decision-makers. Future studies should address clinically important outcomes for clinicians and decision-makers more often, such as the need for neonatal intensive care unit treatment, neonatal mortality, and later health and development of the offspring. Further studies should also aim to address important outcomes for parents, such as infant growth, sleep, behavior, or overall well-being of the infant. Furthermore, the studies could utilize placebo-controlling for intravenous infusion and oral tablets to further improve the quality of the studies, meaning that the other group would get an intravenous iron infusion followed by placebo tablets and the other group would receive an intravenous placebo infusion followed by iron tablets, as this would enable a true masking for all participants and trial assessors. Thus, large-scale quality studies both from high-income and LMIC countries are still needed. The correlation between maternal iron status and neonatal iron status warrants still further investigation.

Oral iron supplementation is sufficient in most cases of pregnancy-related IDA, but the rapid increase in hemoglobin with intravenous preparations can be beneficial when IDA is diagnosed late in pregnancy. However, the optimal timing of intravenous administration is not yet understood well—whether the earlier treatment would provide better results, or whether a rapid treatment in late pregnancy would be effective enough. However, certain precautions and clear thresholds for intravenous iron therapy are needed due to the U-shaped curve of neonatal outcomes [11], and the higher umbilical ferritin levels intravenous therapy seems to achieve. The current evidence does not provide clear answers on how ID during pregnancy should be treated.

## Conclusion

Intravenous iron supplementation had similar effectiveness on neonatal outcomes as oral iron supplementation during pregnancy. Based on these results, both strategies are possible for the treatment of maternal iron deficiency anemia, when the interest is in the well-being of the fetus and neonate. The evidence was limited due to risk of bias and the low number of the included original studies and; thus, further evidence is needed before giving strong recommendations in either direction.

## Supplementary Information

Below is the link to the electronic supplementary material.

ESM 1 (DOCX 1.44 MB)

## Data Availability

All data generated during the review process available upon request from the corresponding author.
