# Supplementary material for: The effect of intravenous iron supplementation compared to oral iron supplementation during pregnancy on neonatal outcomes—a systematic review of randomized controlled trials
Source: Eur J Pediatr. 2025 Oct 13;184(11):684. doi: 10.1007/s00431-025-06522-w (PMC12518418; doi:10.1007/s00431-025-06522-w)

**Supplementary materials**

[**Complete search strategy** 2](#_Toc191560089)

[**PRISMA Checklist** 3](#_Toc191560090)

[**Table S1:** Characteristics of the included studies 6](#_Toc191560091)

[**Table S2** Study participant baseline characteristics. 8](#_Toc191560092)

[**Figure S1:** PRISMA flowchart of the study selection process 10](#_Toc191560093)

[**Figure S2** Risk of bias in the included studies for objective outcomes. 11](#_Toc191560094)

[**Figure S3** Risk of bias of the included studies for subjective outcomes. 12](#_Toc191560095)

[**Figure S4** Sensitivity analysis with only low risk of bias studies included for the mean gestation length 13](#_Toc191560096)

[**Figure S5** Stratified analysis of high-income vs low-income countries for the mean gestation length 14](#_Toc191560097)

[**Figure S6** Funnel plot to detect publication bias for the mean gestation length 15](#_Toc191560098)

[**Figure S7** Sensitivity analysis with high risk of bias studies removed for the preterm birth rate 16](#_Toc191560099)

[**Figure S8** Stratified analysis of high-income vs low-income countries for the preterm birth rate 17](#_Toc191560100)

[**Figure S9** Funnel plot to detect publication bias for the preterm birth rate 18](#_Toc191560101)

[**Figure S10** Sensitivity analysis with low risk of bias studies included for the birthweight outcome. 19](#_Toc191560102)

[**Figure S11** Stratified analysis of high-income vs low- and middle-income countries for the birthweight 20](#_Toc191560103)

[**Figure S12** Funnel plot to detect publication bias for the birthweight 21](#_Toc191560104)

[**Figure S13** Sensitivity analysis with low risk of bias studies included for the cord blood hemoglobin outcome. 22](#_Toc191560105)

[**Figure S14** Sensitivity analysis with low risk of bias studies included for the cord blood ferritin outcome. 23](#_Toc191560106)

[**Figure S15** Funnel plot to detect publication bias for the cord blood hemoglobin 24](#_Toc191560107)

[**Figure S16** Funnel plot to detect publication bias for the cord blood ferritin 25](#_Toc191560108)

# **Complete search strategy**

Date of search: original November 15, 2024, updated February 10, 2025.

**Pubmed**

Search phrase: Intravenous AND iron AND pregnancy

("intraveneous"[All Fields] OR "intraveneously"[All Fields] OR "intravenous"[All Fields] OR "intravenously"[All Fields]) AND ("iron"[MeSH Terms] OR "iron"[All Fields]) AND ("pregnancy"[MeSH Terms] OR "pregnancy"[All Fields] OR "pregnancies"[All Fields] OR "pregnancy s"[All Fields])

**Translations**

intravenous: "intraveneous"[All Fields] OR "intraveneously"[All Fields] OR "intravenous"[All Fields] OR "intravenously"[All Fields]

iron: "iron"[MeSH Terms] OR "iron"[All Fields]

pregnancy: "pregnancy"[MeSH Terms] OR "pregnancy"[All Fields] OR "pregnancies"[All Fields] OR "pregnancy's"[All Fields]

**Scopus:**

Search phrase: TITLE-ABS-KEY ( intravenous AND iron AND pregnancy )

# **PRISMA Checklist**

| **Section and Topic** | **Item #** | **Checklist item** | **Location where item is reported** |
| --- | --- | --- | --- |
| **TITLE** | | |  |
| Title | 1 | Identify the report as a systematic review. | 1 |
| **ABSTRACT** | | |  |
| Abstract | 2 | See the PRISMA 2020 for Abstracts checklist. | 2 |
| **INTRODUCTION** | | |  |
| Rationale | 3 | Describe the rationale for the review in the context of existing knowledge. | 4 |
| Objectives | 4 | Provide an explicit statement of the objective(s) or question(s) the review addresses. | 4 |
| **METHODS** | | |  |
| Eligibility criteria | 5 | Specify the inclusion and exclusion criteria for the review and how studies were grouped for the syntheses. | 5 |
| Information sources | 6 | Specify all databases, registers, websites, organisations, reference lists and other sources searched or consulted to identify studies. Specify the date when each source was last searched or consulted. | 5 |
| Search strategy | 7 | Present the full search strategies for all databases, registers and websites, including any filters and limits used. | 5 |
| Selection process | 8 | Specify the methods used to decide whether a study met the inclusion criteria of the review, including how many reviewers screened each record and each report retrieved, whether they worked independently, and if applicable, details of automation tools used in the process. | 5-6 |
| Data collection process | 9 | Specify the methods used to collect data from reports, including how many reviewers collected data from each report, whether they worked independently, any processes for obtaining or confirming data from study investigators, and if applicable, details of automation tools used in the process. | 5-6 |
| Data items | 10a | List and define all outcomes for which data were sought. Specify whether all results that were compatible with each outcome domain in each study were sought (e.g. for all measures, time points, analyses), and if not, the methods used to decide which results to collect. | 5 |
|  | 10b | List and define all other variables for which data were sought (e.g. participant and intervention characteristics, funding sources). Describe any assumptions made about any missing or unclear information. | 5 |
| Study risk of bias assessment | 11 | Specify the methods used to assess risk of bias in the included studies, including details of the tool(s) used, how many reviewers assessed each study and whether they worked independently, and if applicable, details of automation tools used in the process. | 6 |
| Effect measures | 12 | Specify for each outcome the effect measure(s) (e.g. risk ratio, mean difference) used in the synthesis or presentation of results. | 6 |
| Synthesis methods | 13a | Describe the processes used to decide which studies were eligible for each synthesis (e.g. tabulating the study intervention characteristics and comparing against the planned groups for each synthesis (item #5)). | 6-7 |
|  | 13b | Describe any methods required to prepare the data for presentation or synthesis, such as handling of missing summary statistics, or data conversions. | 6-7 |
|  | 13c | Describe any methods used to tabulate or visually display results of individual studies and syntheses. | 6-7 |
|  | 13d | Describe any methods used to synthesize results and provide a rationale for the choice(s). If meta-analysis was performed, describe the model(s), method(s) to identify the presence and extent of statistical heterogeneity, and software package(s) used. | 6-7 |
|  | 13e | Describe any methods used to explore possible causes of heterogeneity among study results (e.g. subgroup analysis, meta-regression). | 6-7 |
|  | 13f | Describe any sensitivity analyses conducted to assess robustness of the synthesized results. | 6-7 |
| Reporting bias assessment | 14 | Describe any methods used to assess risk of bias due to missing results in a synthesis (arising from reporting biases). | 6-7 |
| Certainty assessment | 15 | Describe any methods used to assess certainty (or confidence) in the body of evidence for an outcome. | 6-7 |
| **RESULTS** | | |  |
| Study selection | 16a | Describe the results of the search and selection process, from the number of records identified in the search to the number of studies included in the review, ideally using a flow diagram. | 8 |
|  | 16b | Cite studies that might appear to meet the inclusion criteria, but which were excluded, and explain why they were excluded. | 8 |
| Study characteristics | 17 | Cite each included study and present its characteristics. | 8 |
| Risk of bias in studies | 18 | Present assessments of risk of bias for each included study. | 8 |
| Results of individual studies | 19 | For all outcomes, present, for each study: (a) summary statistics for each group (where appropriate) and (b) an effect estimate and its precision (e.g. confidence/credible interval), ideally using structured tables or plots. | 8-9 |
| Results of syntheses | 20a | For each synthesis, briefly summarise the characteristics and risk of bias among contributing studies. | 8-9 |
|  | 20b | Present results of all statistical syntheses conducted. If meta-analysis was done, present for each the summary estimate and its precision (e.g. confidence/credible interval) and measures of statistical heterogeneity. If comparing groups, describe the direction of the effect. | 8-9 |
|  | 20c | Present results of all investigations of possible causes of heterogeneity among study results. | 8-9 |
|  | 20d | Present results of all sensitivity analyses conducted to assess the robustness of the synthesized results. | 8-9 |
| Reporting biases | 21 | Present assessments of risk of bias due to missing results (arising from reporting biases) for each synthesis assessed. | 8-9 |
| Certainty of evidence | 22 | Present assessments of certainty (or confidence) in the body of evidence for each outcome assessed. | 8-9 |
| **DISCUSSION** | | |  |
| Discussion | 23a | Provide a general interpretation of the results in the context of other evidence. | 10-12 |
|  | 23b | Discuss any limitations of the evidence included in the review. | 10-12 |
|  | 23c | Discuss any limitations of the review processes used. | 10-12 |
|  | 23d | Discuss implications of the results for practice, policy, and future research. | 10-12 |
| **OTHER INFORMATION** | | |  |
| Registration and protocol | 24a | Provide registration information for the review, including register name and registration number, or state that the review was not registered. | 1 |
|  | 24b | Indicate where the review protocol can be accessed, or state that a protocol was not prepared. | 1 |
|  | 24c | Describe and explain any amendments to information provided at registration or in the protocol. | 11 |
| Support | 25 | Describe sources of financial or non-financial support for the review, and the role of the funders or sponsors in the review. | 1 |
| Competing interests | 26 | Declare any competing interests of review authors. | 1 |
| Availability of data, code and other materials | 27 | Report which of the following are publicly available and where they can be found: template data collection forms; data extracted from included studies; data used for all analyses; analytic code; any other materials used in the review. | 1 |

# **Table S1:** Characteristics of the included studies

| **Study** | **Country** | **Income status** | **Blinding** | **Study period** | **Anemia** | **Intervention** | **Control** | **Funding** | **COI** |
| --- | --- | --- | --- | --- | --- | --- | --- | --- | --- |
| Afolabi et al 2024 | Nigeria | Low | No | 2021–2023 | All anemia | single infusion of ferric carboxymaltose  (20 mg/kg to a maximum total dose of 1000 mg) | oral ferrous sulphate  (200mg tablets containing 65mg elemental iron three  times daily until 6 weeks after delivery | Reported, non-corporate | Reported, none |
| Al et al 2005 | Turkey | Middle | No | 2004 | Iron-deficiency | iron sucrose dose: weight (target hemoglobin – actual hemoglobin) x 0.24 x 500 mg, rounded up to the nearest multiple of 100 mg. | three 100-mg iron tablets per day (100mg elemental iron three times per day) | Not reported | Not reported |
| Awomolo et al 2023 | USA | High | No | 2018–2021 | All anemia | 2 intravenous infusions of 510mg ferumoxytol administered 3 to 7 days apart | 325 mg oral ferroussulfate, twice daily until delivery. (65mg elemental iron twice daily) | Sponsored by pharmaceuticals | Reported |
| Bayoumeu et al 2002 | France | High | No | - | Iron-deficiency | iron sucrose dose: weight (target hemoglobin – actual hemoglobin) x 0.24 x 500 mg, rounded up to the nearest multiple of 100 mg. Given in 6 days. | three 80mg iron sulfate tablets (ie, a  total of 240 mg of elemental iron per day for 4 weeks). | Not reported | Not reported |
| Bencaiova et al 2009 | Switzerland | High | No | 2003–2005 | No anemia | 2 or 3 doses of 200mg intravenous iron sucrose. | Daily 80mg ferrous sulphate until delivery (elemental iron not specified) | Not reported | Not reported |
| Breymann et al 2016 | Multinational | N/A | No | - | Iron-deficiency | Ferric carboxymaltose total of 1000–1500mg intravenous | Oral ferrous sulfate 200mg daily for 12 weeks (elemental iron not specified) | Sponsored by pharmaceuticals | Reported, industry |
| Chauhan et al 2024 | India | Middle | No | - | All anemia | iron sucrose total dose: 2.4 × (target Hb - actual Hb) × weight (kg) + 500mg | 335mg dried ferrous sulfate with 100mg  elemental iron. Twice daily for 4 weeks and then once daily for 6 months. | No specific funding | Reported, none |
| Derman et al 2025 | India | Middle | No | 2021-2023 | Iron-deficiency | IV ferric carboxymaltose or ferric derisomaltose 20mg/kg max 1000mg single dose | Ferrous suplate 60mg twice daily thoughout pregnancy (elemental iron not specified) | Reported, non-corporate | Reported |
| Gupta et al 2014 | India | Middle | No | 2009–2010 | Iron-deficiency | iron sucrose total dose: 2.4 × (target Hb - actual Hb) × weight (kg) + 500mg | 180mg elemental iron daily for 4 weeks | Not reported | Not reported |
| Khalafallah et al 2010 | Australia | High | No | 2007–2009 | Iron-deficiency | iron polymaltose bodyweight (maximum90 kg) in kg x (target Hb - actual Hb) x constant factor (0.24) + irondepot (500mg) + same oral iron | Iron sulphate 250mg, elemental iron 80mg once daily until delivery. | Reported, non-corporate | Reported, none |
| Khalafallah et al 2018 | Australia | High | No | 2013–2014 | Iron-deficiency | single short infusion of 1000 mg intravenous ferric carboxymaltose or single infusion of intravenous iron polymaltose 1000mg. | Iron sulphate 325mg (elemental iron 105 mg) daily until delivery | Reported, non-corporate | Reported, none |
| Kochar et al 2013 | India | Middle | No | - | Iron-deficiency | Iron sucrose total dose: pre-pregnancy bodyweight x Hb deficit × 0.24 + 500 mg | Ferrous suplhate 200mg (elementral iron 60mg) three tablets per day for 4 weeks | Not reported | Reported, none |
| Neogi et al 2019 | India | Middle | No | 2014–2017 | Iron-deficiency | Iron sucrose total dose: bodyweight x hemoglobin deficit x 0.3 + bodyweight x10. | 100mg elemental rion twice a day until 6 weeks postpartum | Reported, non-corporate | Reported, none |
| Pasricha et al 2023 | Malawi | Low | No | 2018–2021 | All anemia | Ferric carboxymaltose 1000mg intravenous | Elemental iron 60mg twice a daily for 90 days. | Reported, non-corporate | Reported |
| Paricha et al 2025 | Malawi | Low | No | 2021-2023 | All anemia | Ferric carboxymaltose 1000mg intravenous | Elemental iron 60mg twice a daily for 90 days. | Reported, non-corporate | Reported |

# **Table S2** Study participant baseline characteristics.

| **Study** | **N of participants** | | **Age in years** | | **Parity (%)** | | **Anemia status  (Hb g/dl, ferritin µg/l)** | |
| --- | --- | --- | --- | --- | --- | --- | --- | --- |
|  | **IV** | **PO** | **IV** | **PO** | **IV** | **PO** | **IV** | **PO** |
| Afolabi et al 2024 | 527 | 529 | median 28 | median 28 | Nulliparous 36% | Nulliparous 35% | mean Hb 9.14 (SD 0.70) mean ferritin 66.4 (SD 108.1) | mean Hb 9.15 (SD 0.69) mean ferritin 77.1 (SD 107.8) |
| Al et al 2005 | 45 | 45 | mean 25 | mean 27 | nulliparous 62% | Nulliparous 42% | mean Hb 9.9 (SD 0.5) mean ferritin 4.1 (SD 2.5) | mean Hb 9.8 (SD 0.6) mean ferritin 5 (SD 2.2) |
| Awomolo et al 2023 | 62 | 62 | mean 27 | mean 27 | Nulliparous 39% | Nulliparous 39% | - | - |
| Bayoumeu et al 2002 | 24 | 23 | mean 25 | mean 25 | Nulliparous 42% | Nulliparous 44% | mean Hb 9.6 (SD 0.8) mean ferritin 6.5 (SD 2.5) | mean Hb 9.7 (SD 0.5) mean ferritin 8.0 (SD 4.0) |
| Bencaiova et al 2009 | 130 | 130 | mean 29 | mean 28 | Mean G2.5P1.9 | Mean G2.5P2.0 | - | - |
| Breymann et al 2016 | 123 | 124 | median 31 | median 31 | - | - | mean Hb 9.8 mean ferritin 12.2 | mean Hb 9.9 mean ferritin 12.4 |
| Chauhan et al 2024 | 134 | 134 | mean 27 | mean 26 | - | - | mean Hb 8.5 mean ferritin 17.8 | mean Hb 8.6 mean ferritin 16.9 |
| Derman et al 2025 | 1456 | 3008 | - | - | Nulliparous  40.1% | Nulliparous  40.8% | mean Hb 9.0 (SD 0.8) mean ferritin 9.4 | mean Hb 9.0 (SD 0.8) mean ferritin 9.5 |
| Gupta et al 2014 | 50 | 50 | mean 25 | mean 25 | Nulliparous 18% | Nulliparous 20% | mean Hb 7.8 (SD 0.4) mean ferritin 10.7 (SD 1.5) | mean Hb 7.9 (SD 0.4) mean ferritin 10.4 (SD 1.9) |
| Khalafallah et al 2010 | 98 | 98 | mean 28 | mean 28 | - | - | mean Hb 10.7 (SD 0.5) mean ferritin 18.1 (SD 16.3) | mean Hb 10.9 (SD 0.5) mean ferritin 17.7 (SD 17.7) |
| Khalafallah et al 2018 | 165 | 81 | mean 29 | mean 29 | - | - | mean Hb 11.4 mean ferritin 12.5 | mean Hb 11.5 mean ferritin 12.5 |
| Kochar et al 2013 | 50 | 50 | mean 24 | mean 23 | - | - | mean Hb 7.7 (SD 0.5) mean ferritin 18.1 (SD 4.6) | mean Hb 7.6 (SD 0.8) mean ferritin 16.5 (SD 5.9) |
| Neogi et al 2019 | 983 | 1016 | mean 25 | mean 24 | Nulliparous 33% | Nulliparous 37% | mean Hb 7.7 | mean Hb 7.7 |
| Pasricha et al 2023 | 430 | 432 | mean 22 | mean 23 | Nulliparous 54% | Nulliparous 56% | mean Hb 8.8 (SD 1.3) median ferritin 26 (IQR 10-81) | mean Hb 8.8 (SD 1.2) median ferritin 28 (IQR 10-63) |
| Pasricha et al 2025 | 297 | 292 | mean 25 | mean 25 | - | - | mean Hb 9.3 (SD 1.3) median ferritin 13 (IQR 9-21) | mean Hb 9.4 (SD 1.3) median ferritin 12 (8-17) |

# **Figure S1:** PRISMA flowchart of the study selection process

**Identification of studies via databases and registers**

Records removed *before screening*:

Duplicate records removed (n = 337)

Record marked ineligible by automation tools (n = 284)

Records identified from:

PubMed (n = 536)

Scopus (n = 460)

**Identification**

Records screened

(n = 375)

Records excluded

(n = 341)

Reports sought for retrieval

(n = 34)

Reports not retrieved

(n = 0)

**Screening**

Reports assessed for eligibility

(n = 34)

Reports excluded:
Wrong outcomes (n = 11)
Wrong patients (n = 4)
Wrong comparator (n = 2)
Protocol (n = 1)
Retracted study (n = 1)

Reports included in review

(n = 15)

**Included**

# **Figure S2** Risk of bias in the included studies for objective outcomes.

**
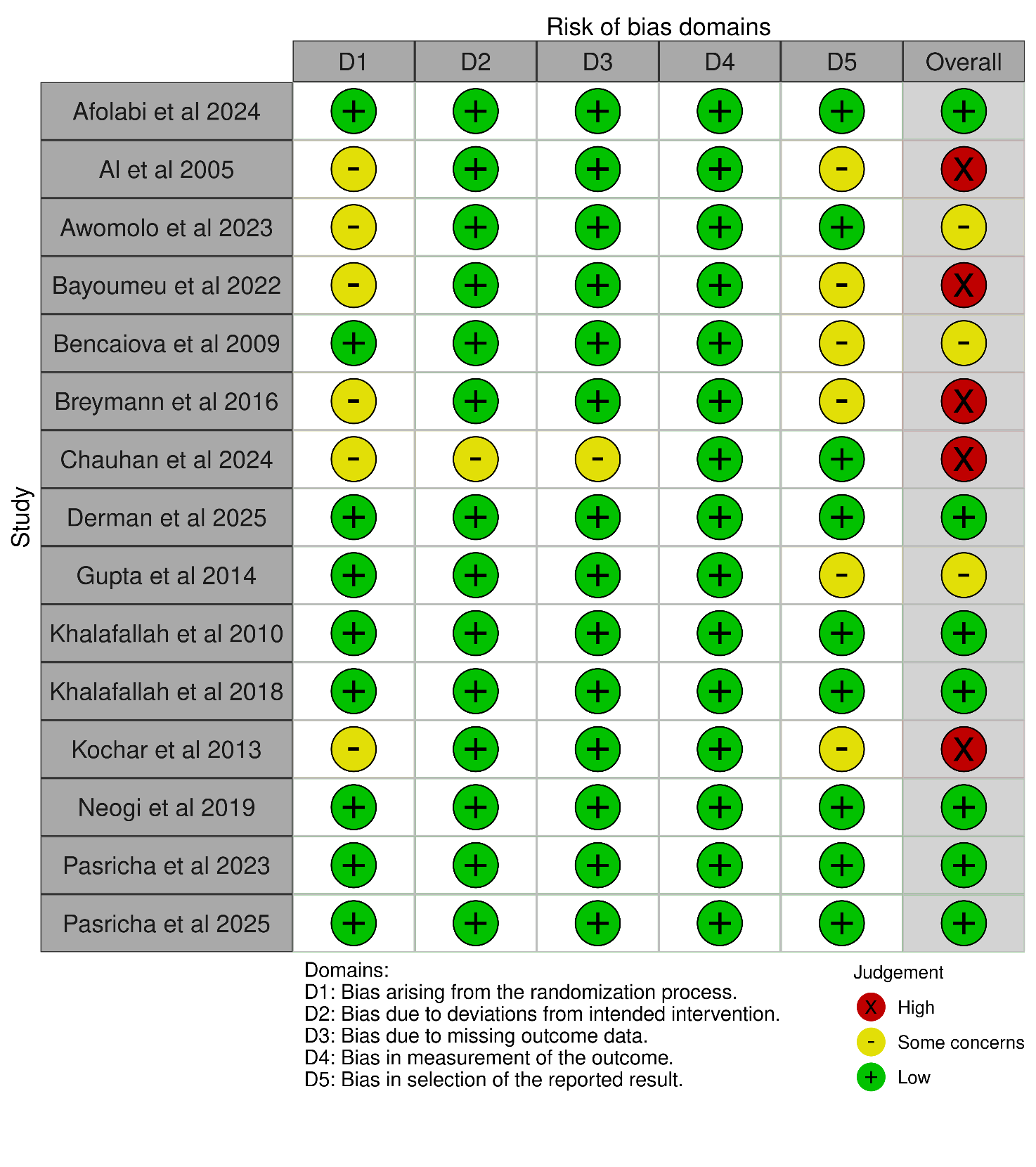
**

# **Figure S3** Risk of bias of the included studies for subjective outcomes.


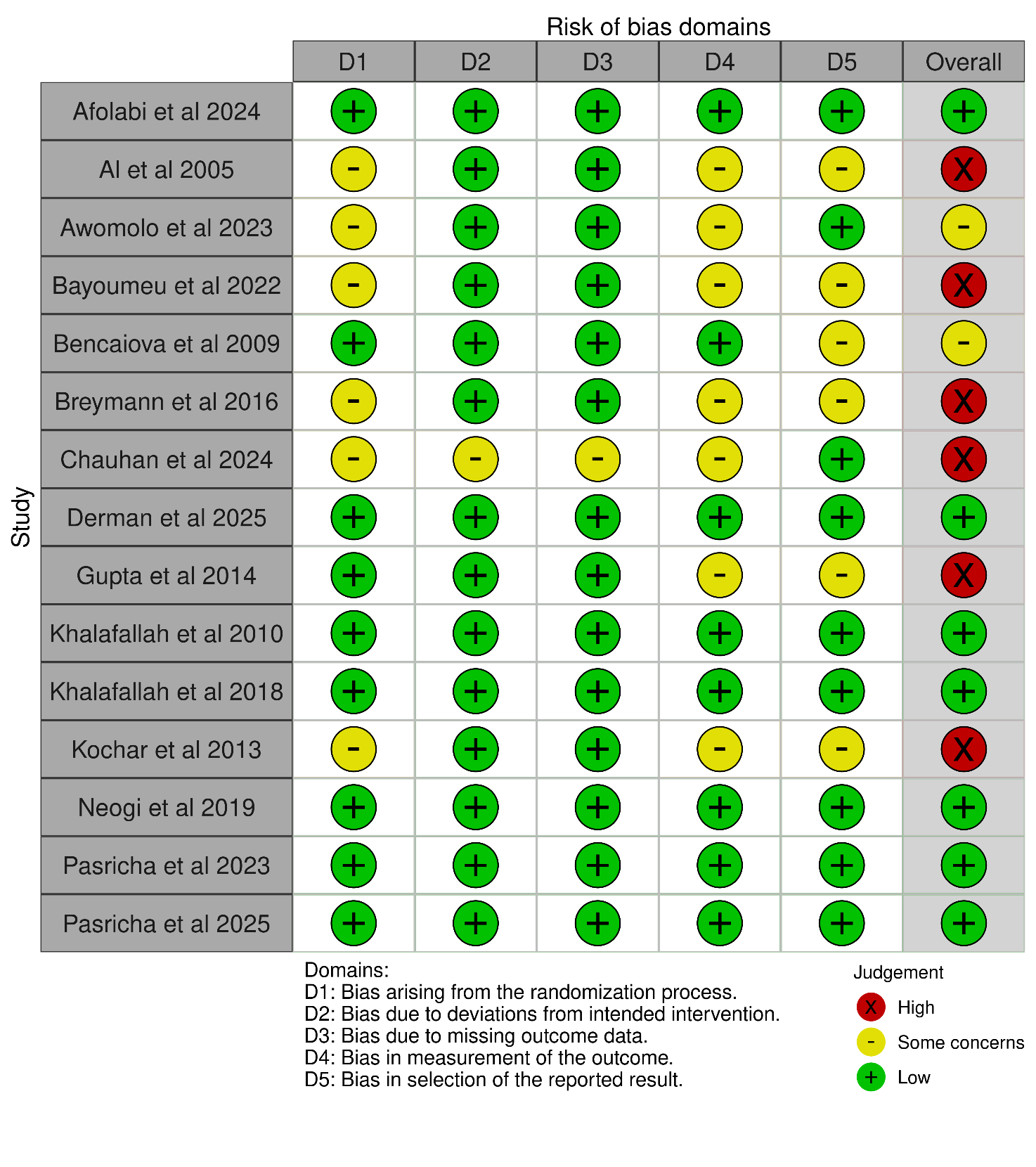


# **Figure S4** Sensitivity analysis with only low risk of bias studies included for the mean gestation length


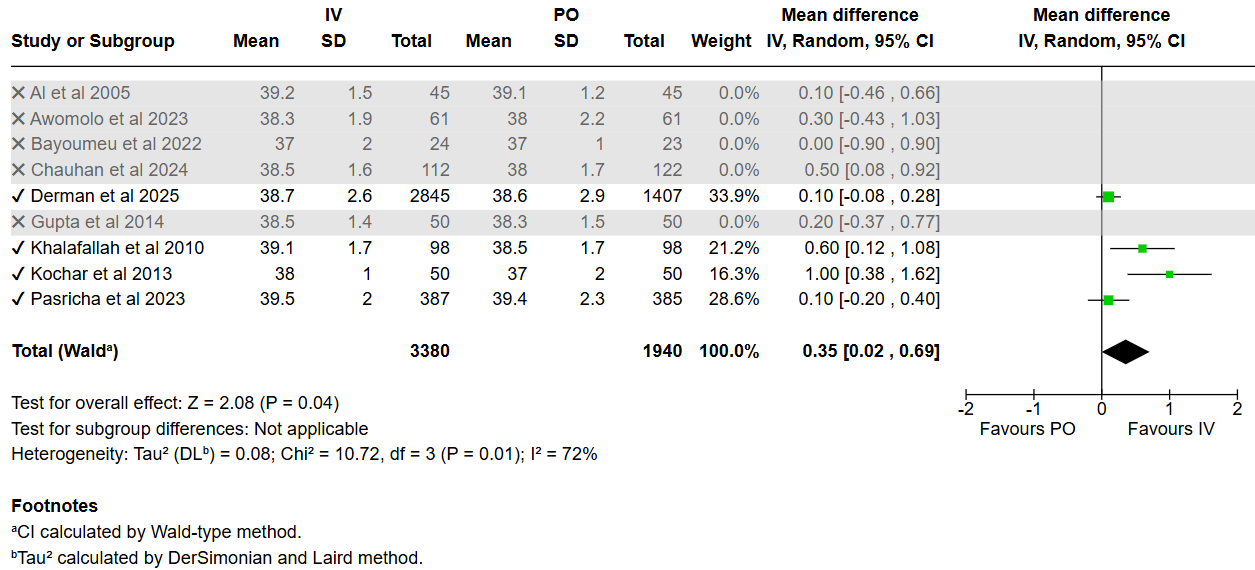


# **Figure S5** Stratified analysis of high-income vs low-income countries for the mean gestation length


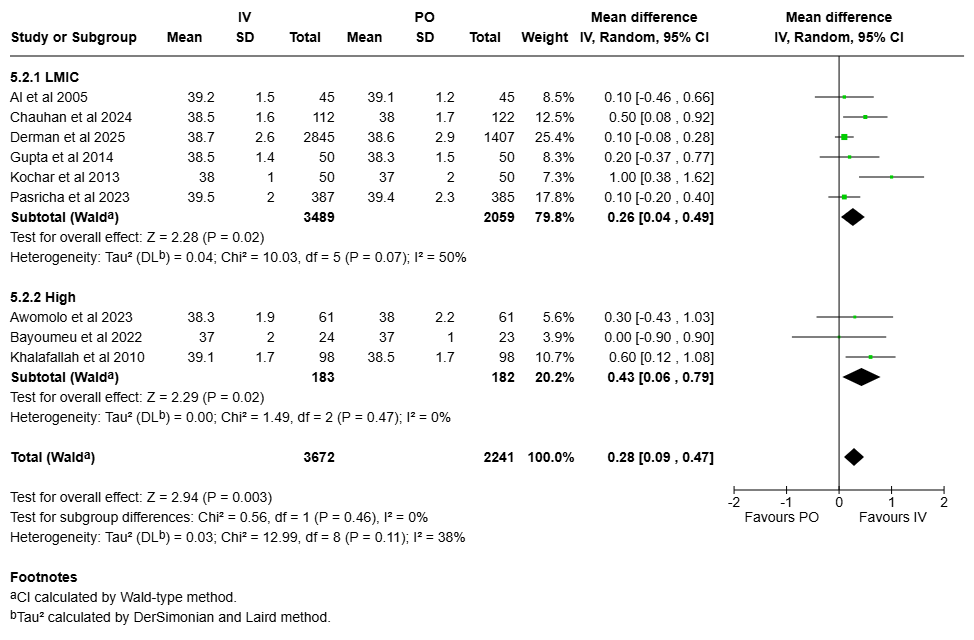


# **Figure S6** Funnel plot to detect publication bias for the mean gestation length


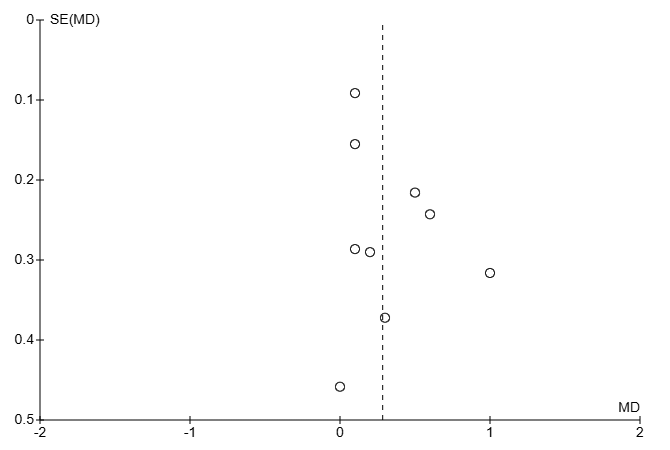


# **Figure S7** Sensitivity analysis with high risk of bias studies removed for the preterm birth rate


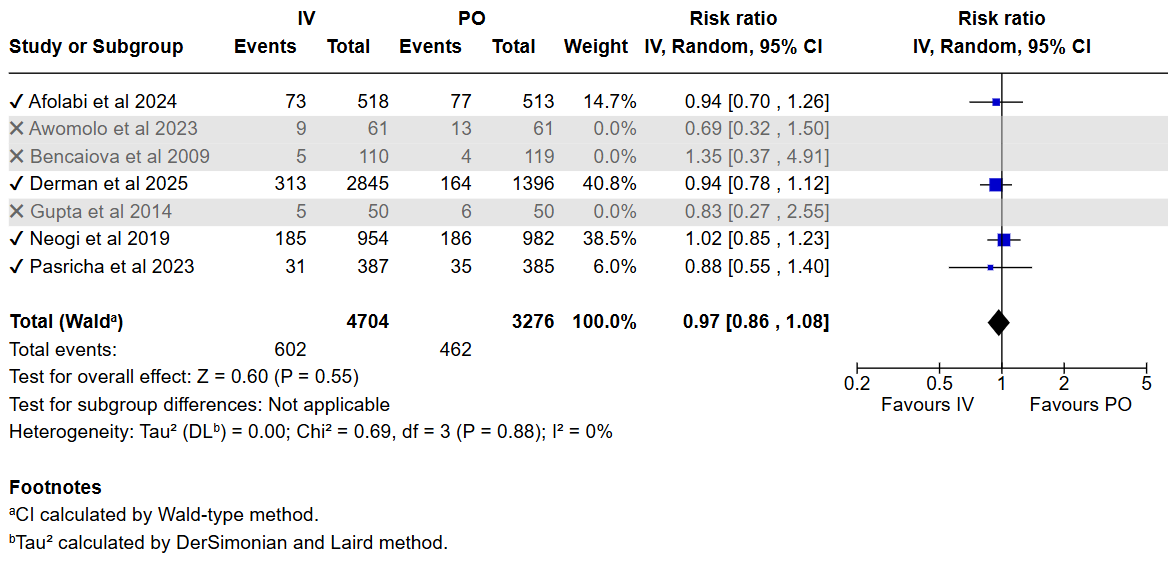


# **Figure S8** Stratified analysis of high-income vs low-income countries for the preterm birth rate


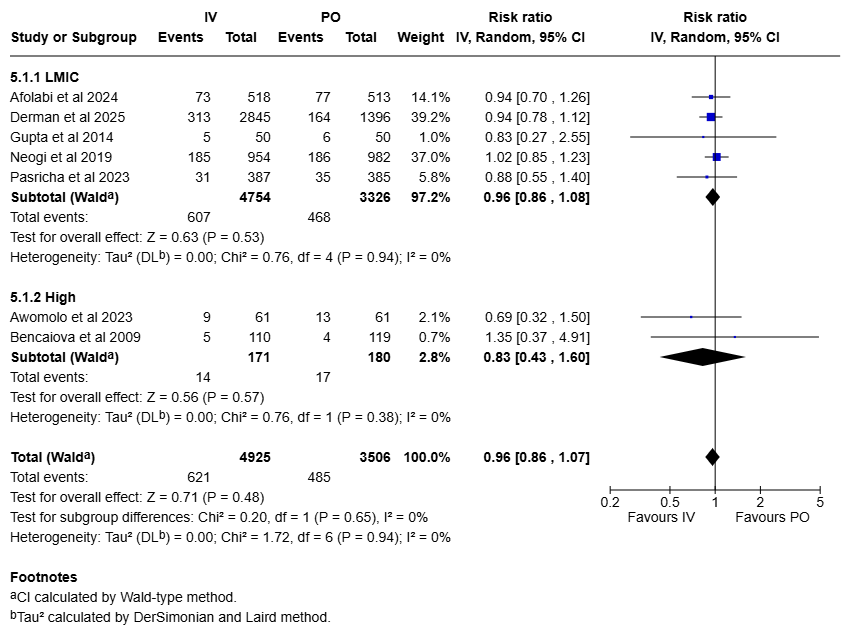


# **Figure S9** Funnel plot to detect publication bias for the preterm birth rate


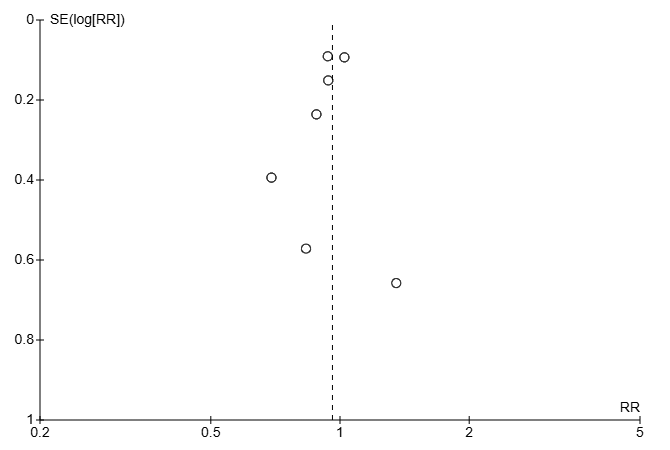


# **Figure S10** Sensitivity analysis with low risk of bias studies included for the birthweight outcome.


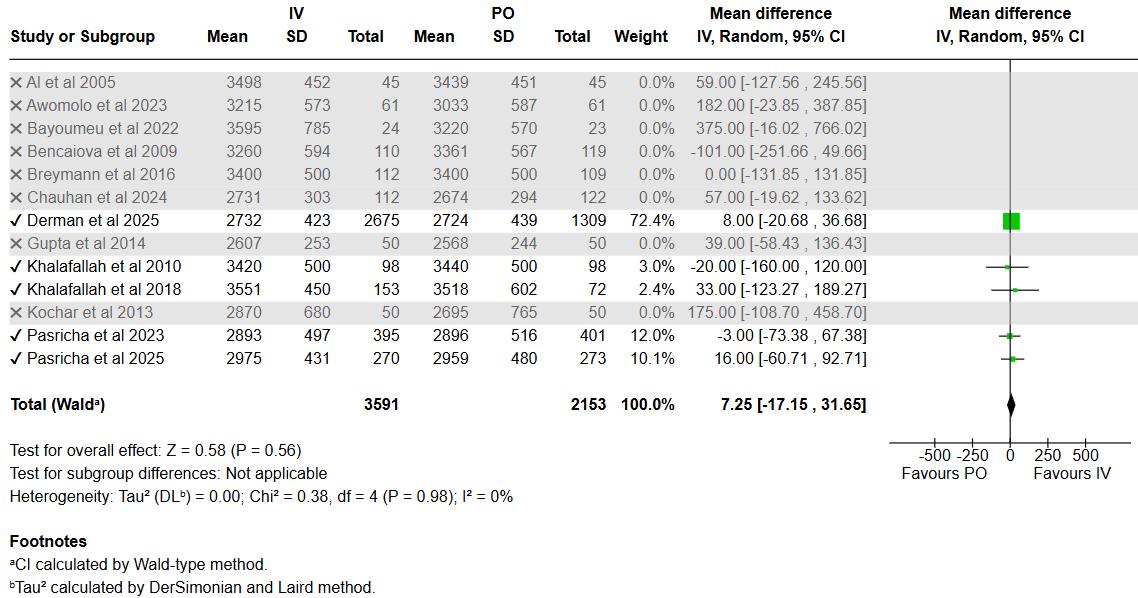


# **Figure S11** Stratified analysis of high-income vs low- and middle-income countries for the birthweight


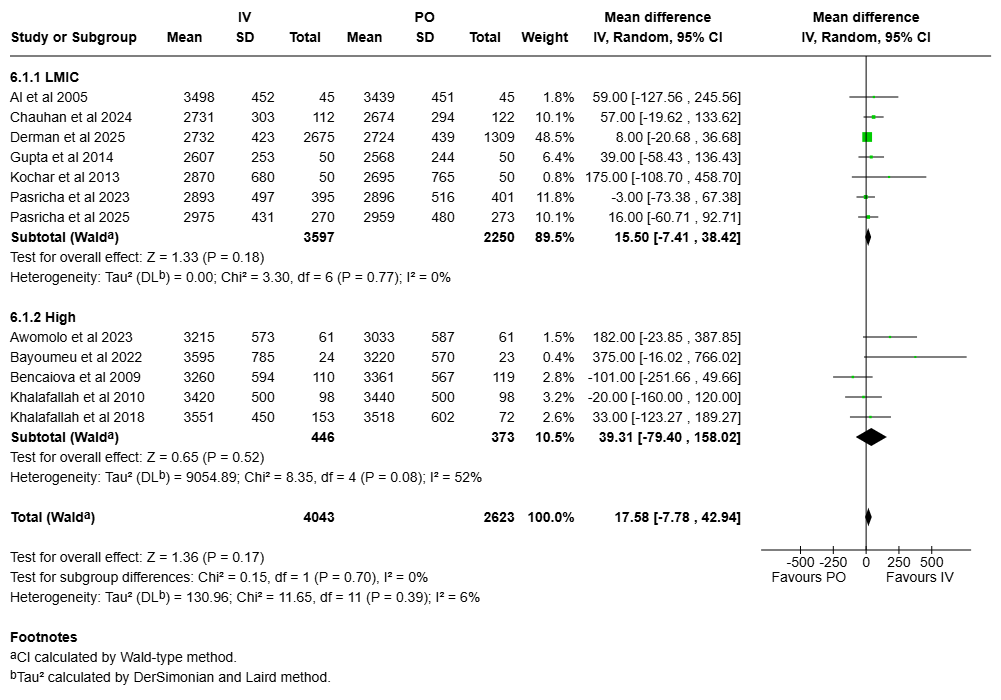


# **Figure S12** Funnel plot to detect publication bias for the birthweight


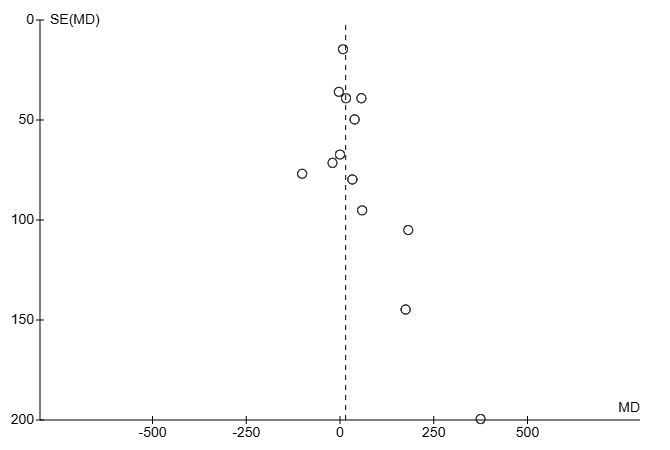


# **Figure S13** Sensitivity analysis with low risk of bias studies included for the cord blood hemoglobin outcome.


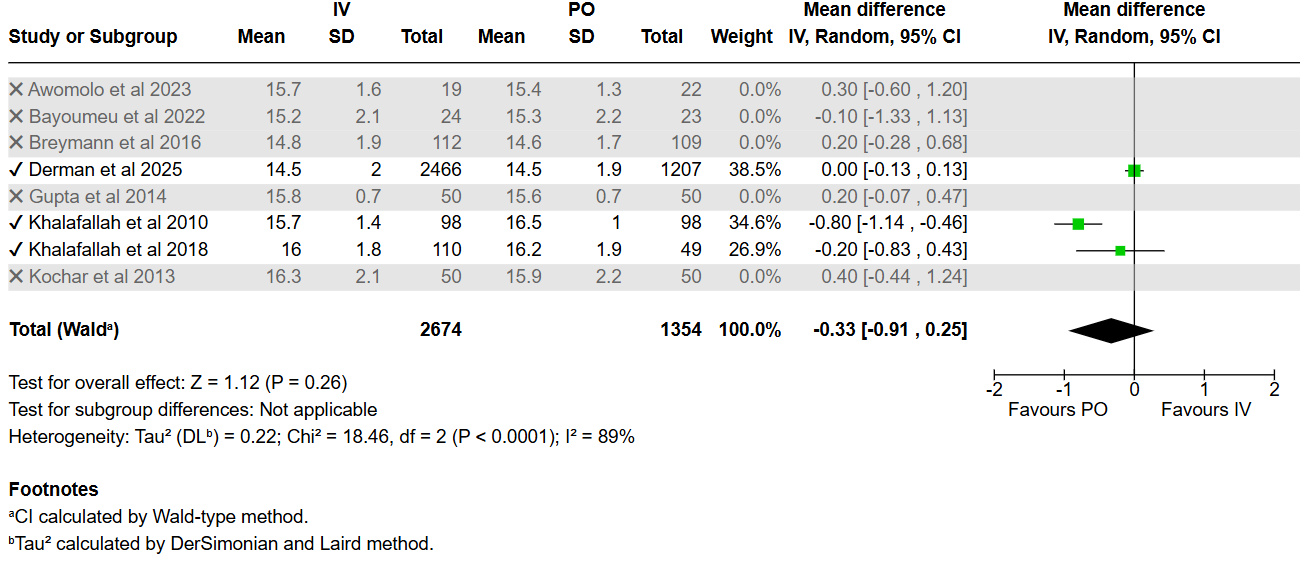


# **Figure S14** Sensitivity analysis with low risk of bias studies included for the cord blood ferritin outcome.


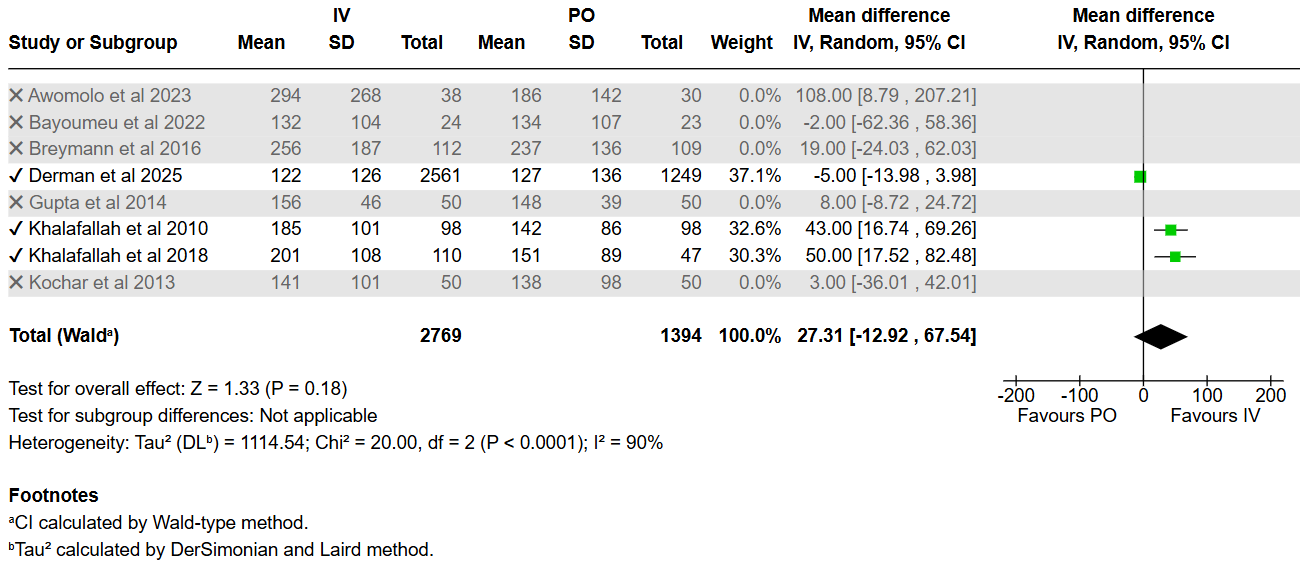


# **Figure S15** Funnel plot to detect publication bias for the cord blood hemoglobin


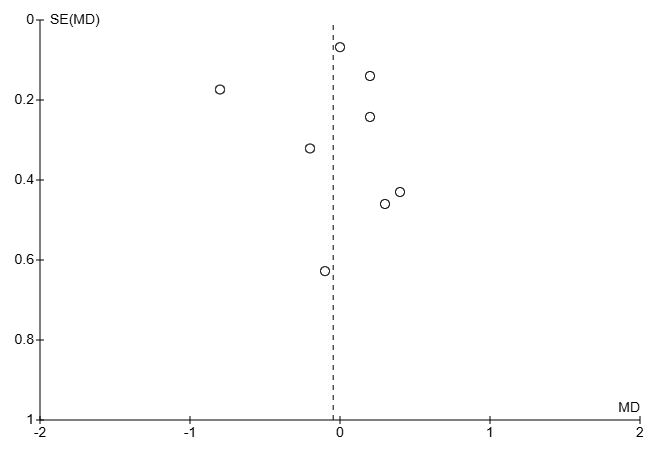


# **Figure S16** Funnel plot to detect publication bias for the cord blood ferritin


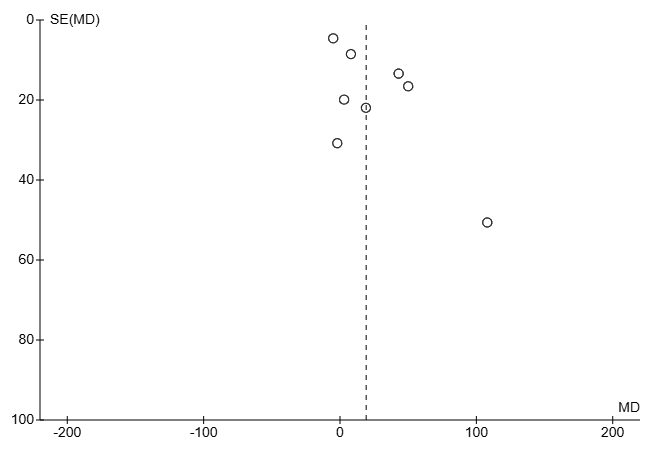

Supplement: Supplementary file 1 — (DOCX 1.44 MB) [file 431_2025_6522_MOESM1_ESM.docx]
